# The Role of Clinicians’ Self-Efficacy in Exercise Promotion for Adults with Venous Leg Ulcers: A Cross-Sectional Study

**DOI:** 10.1177/15347346251321550

**Published:** 2025-02-25

**Authors:** Jane O’Brien, Damhnat McCann, Christina N Parker, Kathleeen Finlayson, Andrew Jull

**Affiliations:** 1 110544 Faculty of Health, School of Nursing, Queensland University of Technology, Victoria Park Rd, Kelvin Grove, QLD 4059, Australia; 2 589598 Centre for Healthcare Transformation, Queensland University of Technology, Brisbane, Australia; 3 60119 College of Health and Medicine, School of Nursing, University of Tasmania, Launceston, Australia; 4 62710 Faculty of Medical & Health Sciences, School of Nursing, University of Auckland, Auckland, New Zealand; 5 Centre for Translational Research in Health (TRANSFORM), University of Auckland, Auckland, New Zealand

**Keywords:** vascular < Diagnosis, venous < Lower extremity wound, wound care/dressings/NPWT or TNP < Wound management

## Abstract

**Introduction:**

Physical activity is recognised for its functional, physical, and psychological benefits in managing venous leg ulcers. Despite these advantages, individuals with venous leg ulcers often remain inactive, largely due to fear of exacerbating their condition and a lack of comprehensive guidance. Clinicians play a crucial role in promoting physical activity, providing support to patients to engage in safe and beneficial exercises. This study aimed to assess clinicians’ knowledge, attitudes, and practices toward physical activity in adults with venous leg ulcers to inform clinical practice and the development of targeted physical activity strategies.

**Methods:**

An online questionnaire was developed to assess clinicians’ familiarity with physical activity guidelines, their confidence in recommending physical activity, and the barriers they encountered in practice.

**Results:**

A total of 141 clinicians, predominantly nurses (99%), completed the survey, with 83% reporting awareness of clinical guidelines for venous leg ulcer management. However, only 25% regularly recommended a general increase in physical activity, 27% recommended calf strengthening exercises, and 38% consistently recommended ankle joint mobility exercises. Key barriers; 1) insufficient training to recommend specific exercises, 2) lack of evidence-based information to provide to patients and 3) limited access to exercise specialists for referrals. Statistical modelling showed that self-efficacy significantly influenced recommendations for ankle mobility exercises; clinicians with higher confidence were 7.6 times more likely to make such recommendations (p < .001). Although attitudes toward prescribing ankle exercises to adults with venous leg ulcers in compression were not statistically significantly related to reported practice (p = .087), they demonstrated relevance to clinical decision making and warrant further investigation.

**Conclusion:**

While clinicians recognise the importance of physical activity for adults with venous leg ulcers, gaps in knowledge, resources, and training limit their ability to provide specific exercise recommendations. Addressing these gaps through large-scale implementation of structured physical activity strategies is essential.

Venous leg ulcers (VLUs) develop as a result of chronic venous insufficiency (CVI),^1^ with the estimated prevalence of VLUs ranging from 1% for adults, 3.6% for individuals over the age of 65,^2^ and 5% for those over 80 years.^3^ VLUs have extended healing periods, with about 60% healing within 6 months and an additional 33% within one year.^4^ Up to 70% of adults with VLUs experience recurrence within three months of wound closure,^4,5^ making VLUs a long-term health issue.^6^ This chronic condition imposes a significant burden in terms of pain^7^ and negatively impacts quality of life,^8^ with approximately 3% of annual healthcare costs dedicated to its direct treatment.^2^

VLU management by clinicians employs a multifaceted strategy to support wound healing, symptom relief, venous return, and to lower recurrence chances. Key approaches include compression therapy, elevating the legs, ankle-focused exercises, and a diet high in protein.^9,10^ Physical activity and/or exercise has substantial functional, physical and psychological benefits for adults with VLUs, enhancing health-related quality of life, physical functioning and improved venous return and ulcer healing rates.^11^

Lower limb exercise is recommended in most international position statements on the care of adults with VLUs^9,12–14^ as an adjunctive treatment of VLUs and evidence extends to clinicians recommending simple progressive and aerobic activity.^11^ There are, however, many barriers for adults with VLUs to participate in PA,^15^ despite known clinical and functional benefits.^11^ Clinicians can play a key role in promoting behaviour change^16^ as are a source of motivation and encouragement for adults with VLU to be physically active on a regular basis.^17^ However, anecdotal evidence suggests PA and or exercise is not well understood by clinicians and not routinely discussed with adults with VLUs. This gap in knowledge may hinder the ability of clinicians to effectively advocate for PA, potentially limiting the overall health benefits that could be gained during this critical period.

A recent small qualitative study highlighted for nurses important strategies including: education of patients, families and clinicians, the provision of consistent and clear exercise advice from clinicians and setting realistic and meaningful goals.^18^ Furthermore, identifying the challenges related to exercise from a clinician's perspective in clinical practice is essential for designing targeted future interventions. The aim of this survey of clinicians therefore was to explore the knowledge, attitude, and practice of clinicians about PA for adults with VLUs.

## Method

### Study Design

An online cross-sectional survey was designed and administered using Survey Monkey,^19^ from November 2020 – March 2021. Ethical approval was granted by the University of Tasmania Human Research Ethics Committee (H0018081). Where applicable, the STROBE cross-sectional checklist was used.^20^

### Survey Development

In the absence of existing survey instruments from the initial literature review, a survey was adapted from prior research that examined obstacles to physician adherence to asthma guideline.^21^ An iterative process of design and refinement was then undertaken by the authors to ensure the survey maintained an appropriate scope. Following development, the survey was pilot tested with six health professionals who were not members of the study team to assess face and content validity and completion time. After incorporating feedback (eg, adjustments to question wording), the survey was completed in consultation with the research team.

### Final Survey

The survey started with a question on whether respondents’ were currently practicing within wound care and treating adults with VLUs to confirm eligibility. The main body of the survey consisted of four sections, which sought clinicians’ views on: familiarity (knowledge), confidence (self-efficacy), barriers to practice and the importance and relevance of PA (agreement and outcome expectancy). The survey used a 5-point Likert scale for respondents to indicate the level of familiarity (1, not at all familiar; 5 extremely familiar), agreement (1, strongly disagree; 5 strongly agree), and self-efficacy (1, not at all confident; 5 extremely confident). A 4-point Likert scale was used for outcome expectancy (1, no effect; 4 large effect) and practice barriers (1, not at all significant; 4 extremely significant). We selected these different response sets based on previous surveys that examined these constructs in relation to guideline adherence.^21^ Awareness was measured using a dichotomous response (yes/no). Demographic data was collected including age, profession, clinical specialisation, length of time working in a wound management setting, highest qualification, membership of specialty wound organisation, percentage of patients treated with an active or past VLU, country of employment, and if practice is predominantly rural or urban. There was one free-text question “*How do you decide which patients may need additional exercises and/or physical activity prescription?*’. The survey was kept short (6-8-min completion time) to encourage response.

The reliability of the survey items was assessed using Cronbach's Alpha. Results indicated that the scales measuring perceived knowledge (familiarity) (α = .867, n = 111), confidence (self-efficacy) (α = .862, n = 116), barriers to practice (α = .891, n = 109) and agreement, (awareness) (α = .901, n = 108) were internally consistent, supporting the validity of the instrument for exploring clinicians’ perspectives on PA for VLUs.

### Population and Sampling

The target population was clinicians (vascular surgeons, dermatologists, nurses, and allied health staff) involved in the care of adults (>18 years of age) with VLUs. Participants were recruited using convenience sampling methods. Two professional associations, mentioned in the Acknowledgements section, assisted by sharing the survey link with their members, followed by a reminder email sent about four weeks later to boost response rates. Additionally, the survey link was shared via the research team's professional and social media networks.

### Study Size

A formal sample size calculation was not performed; instead, the sample size was based on the anticipated reach of professional networks and previous response rates in similar studies.^21^

### Data Management

The survey did not collect any personally identifying information. Measures to prevent multiple submissions were not implemented, as repeat responses were considered unlikely given the survey's length, topic, and absence of participation incentives. To minimise response bias, the survey was designed to be short and accessible, encouraging participation without requiring forced responses (except for eligibility confirmation). Additionally, pilot testing helped refine question clarity and prevent misinterpretation, reducing bias from varying interpretations of questions.

### Consent

The survey introduction explained the study's objectives and clarified that by participating, respondents consented to the use of their deidentified data for analysis and dissemination in research publications and presentations. Given the data's anonymity, participants could not withdraw their responses after completing the survey.

### Statistical Analysis

SPSS 29.0 was used for statistical analysis. Descriptive statistics were used to analyse the data. For each analysis of the four exercise recommendations, our dependent variable of interest was a clinician's self-reported adherence to recommendations. We considered clinicians to be adherent if they reported following a guideline component more than 90% of the time, based on previous definitions of adherence.^22^ We dichotomised the responses. A factor was present if clinicians answered 4 or 5 on a 5-point Likert scale or 3 or 4 on a 4-point Likert scale.

We used chi square analysis to compare each of the independent variables with self-reported adherence to the guideline components. Differences between age groups and numbers of years working in wound management in relation to attitudes, knowledge and practices were examined using t-tests. Relationships between attitudes, knowledge and practices were examined using Spearman's correlation coefficients and chi-test.

The survey data was analysed with descriptive statistics, and due to varying response rates per question, proportions were calculated using only the valid responses for each item. This survey did not use forced-response requirements, except on the first question which was necessary to confirm participant eligibility. All responses provided were used (ie, no case-wise deletion) and no imputation was carried out. The free-text question was analysed using thematic content analysis.^23^

A binary logistic regression analysis was conducted to explore the relationship between independent variables and the dependent variable, whether clinicians recommend ankle exercises for patients with VLUs. Following the removal of variables identified as at risk of multi-collinearity, five variables significantly associated with recommending ankle exercises at the bivariate level were included in the initial model. Assumption testing conducted before the analysis did not indicate any violations. A backwards stepwise process was followed to remove variables one by one which were not related to the outcome and did not contribute significantly to the model.

## Results

One hundred and fifty clinicians responded to the online questionnaire but 9 responded ‘no’ to the eligibility question ‘Are you currently active in clinical practice?’: Hence 141 participants were included in the final analysis. The median length of time working in wound management was 11 years (range 6mths – 40 years). Median age was 50 years (range 21-66). Most of the respondents were from the United Kingdom and Australia (see results in Table 1).

**Table 1.** Characteristics of survey respondents (n = 141)

Characteristics	Count (%)
Type of clinician (*n *= 99)^a^	
Nurse	98 (99.0)
Doctor	-
Physiotherapist	-
Occupational Therapist	-
Other	1(1.0)
Member of Wound Organisation (*n *= 98)^b^	59 (60.0)
Country^c^	
United Kingdom	61 (63.5)
Australia	28 (29.2)
Austria	1 (1.0)
Ukraine	1 (1.0)
Ireland	5 (5.2)
Category of qualification (*n *= 74)^d^	
Bachelor's degree	30 (40.5)
Bachelor's degree with Honours	8 (10.8)
Graduate certificate	7 (9.5)
Graduate diploma	10 (13.5)
Master's level	18 (24.3)
PhD	1 (1.4)
Practice location (*n *= 97)^e^	
Urban	59 (60.8)
Rural	38 (39.2)

^a^ Missing data on qualification: *n* = 51 (34%).

^b^ Missing data on qualification: *n* = 52 (34.7%).

^c^ Missing data on qualification: *n* = 54 (36%).

^d^ Missing data on qualification: *n* = 76 (50.7%).

^e^ Missing data on qualification: *n* = 53 (35.3%).

### Current Practice Recommendations

**Figure 1.**
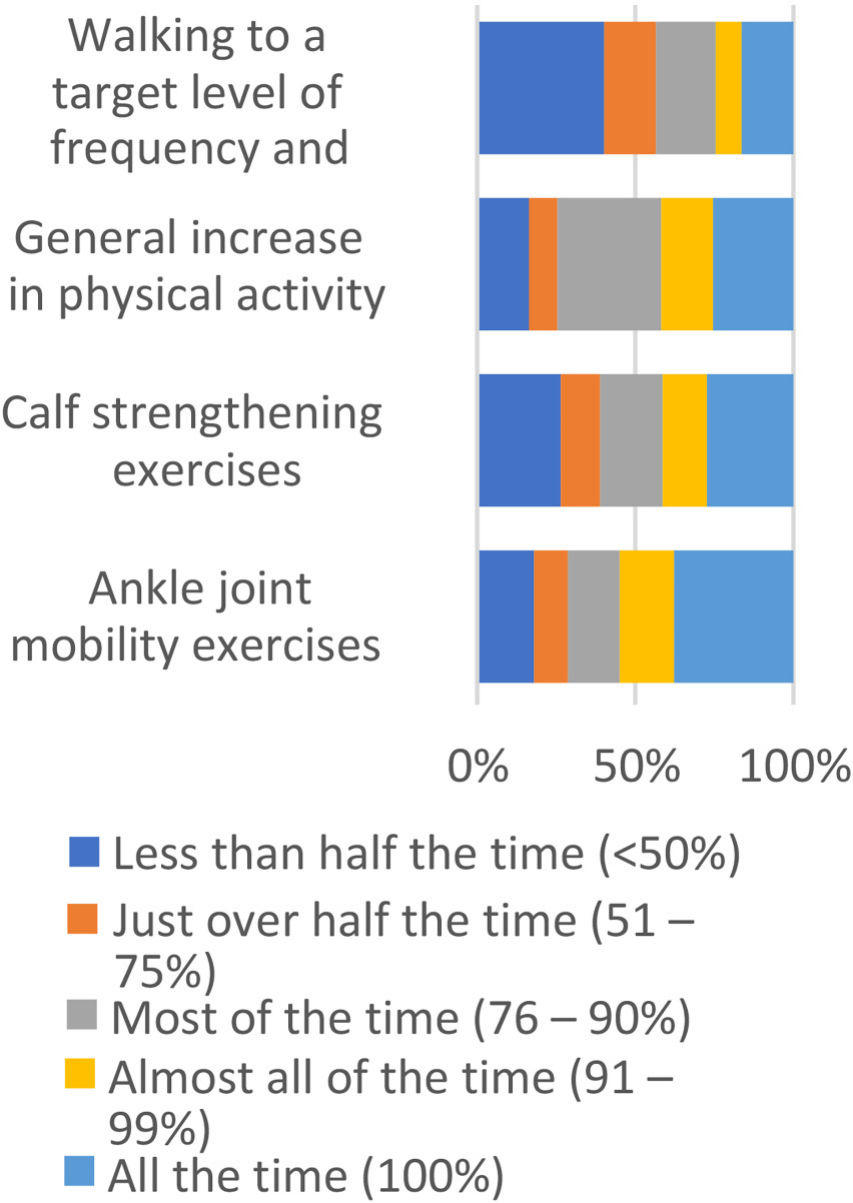
How often clinicians recommend specific types of exercises and walking to patients with VLUs.

The most often recommended exercise ‘all of the time’ by clinicians was ankle joint mobility exercises (38%), followed by calf strengthening exercises, a general increase in PA whereas specificity of walking was only recommended 8% all of the time to patients with VLUs, see Figure 1.

### Perceived Knowledge (Familiarity) in Relation to Exercise Advice for Adults with VLUs

Most respondents reported having at least adequate knowledge in relation to simple exercises that increase ankle joint mobility (66.1%, 74/118) and calf-muscle strength (59.3%, 67/113). Less than half of the respondents (44.7%, 50/112) reported adequate knowledge in relation to the duration and frequency of walking recommended for VLU patients (see Table S1).

### Confidence in Recognising and Managing Adults in Relation to PA (Self-Efficacy)

Most respondents reported moderate confidence in their ability to demonstrate and prescribe ankle joint mobility exercises (68.4%, 80/117) and calf strengthening exercises (70.1%, 82/117) for a VLU patient. Less than half of the respondents were moderately confident (47.5%, 55/116) to provide advice for an inactive adult with a VLU to build towards a targeted increase in walking, although 59% (69/117) reported at least moderate confidence to discuss the evidence for exercises with adults with a VLU (see Table S1).

### Barriers to Assessing and Managing PA in Practice

**Figure 2.**
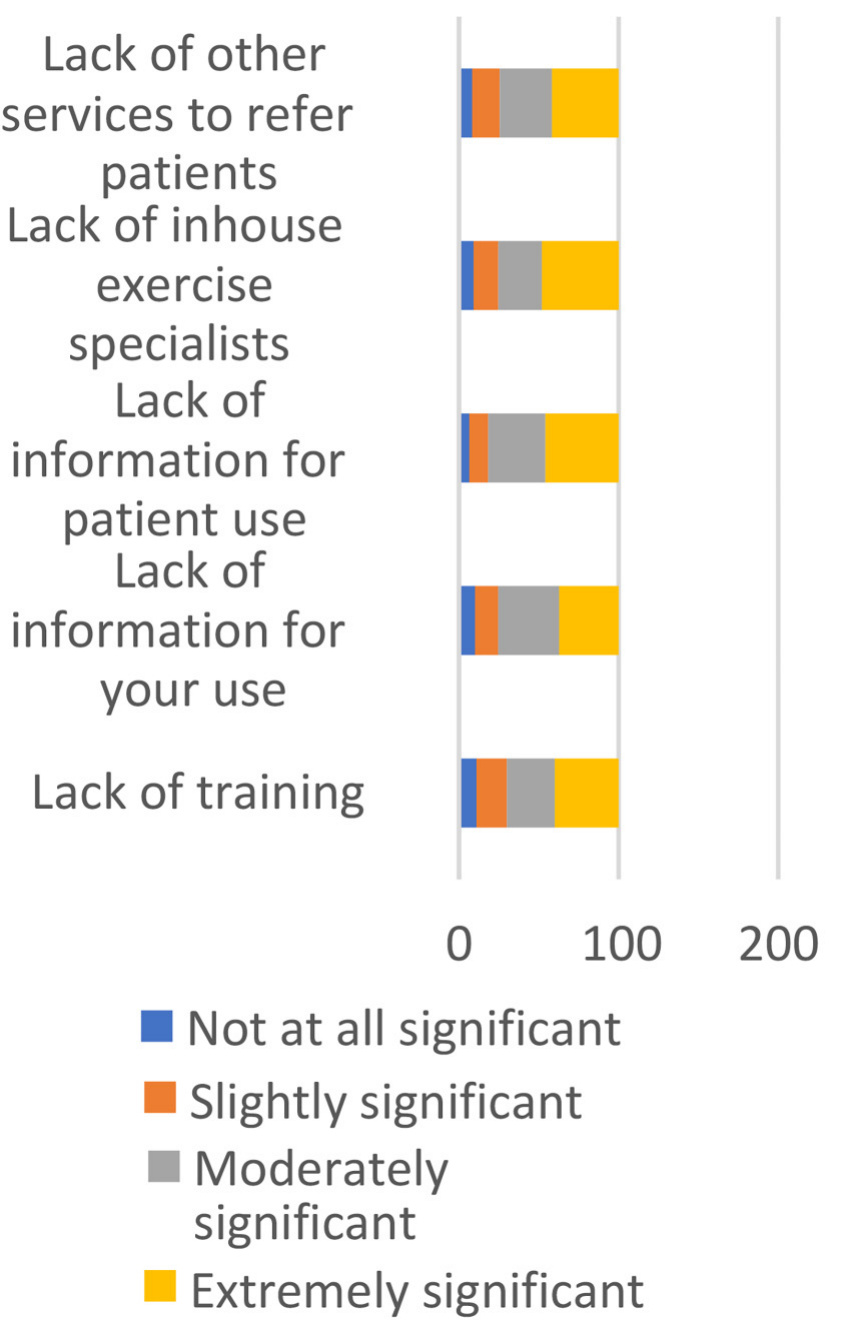
Barriers to practice.

Commonly reported barriers to assessing and managing PA in practice included ‘lack of training’, ‘lack of information for your use’, ‘lack of information for patient use’, ‘lack of inhouse exercise specialists’ and ‘lack of other services to refer patients’. Most respondents reported that ‘lack of training’ (70%, 77/110) and ‘lack of information for your use’ (75.5%, 83/110) was at least moderately significant as a barrier. Almost all participants (81.7%, 89/109) reported that a lack of information for patient use was a barrier, 75.5% (83/110) reported that a lack of exercise specialists was a barrier, and 74.5% (82/110) reported a lack of services to refer to as a barrier to how frequently they recommended exercise (see Figure 2).

### The Importance and Relevance of PA (Agreement, Awareness, and Outcome Expectancy)

**Figure 3.**
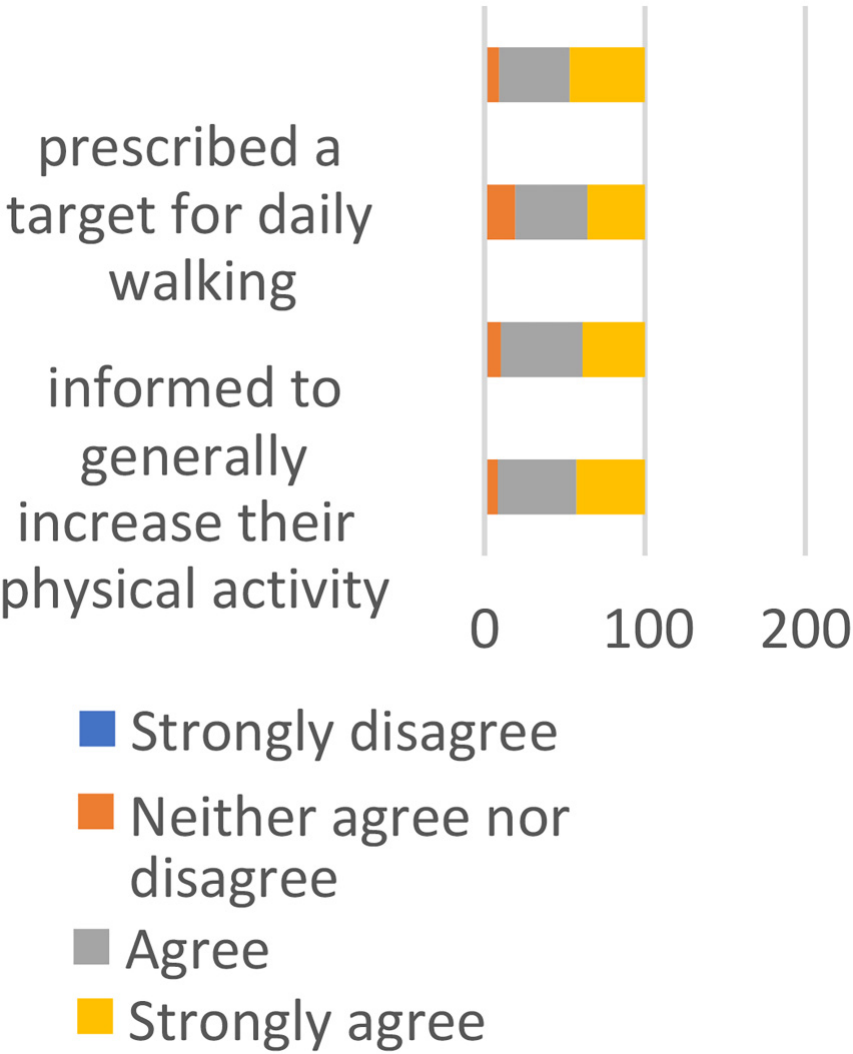
Agreement with the prescriptive statements.

Almost all clinicians agreed that patients with a VLU in compression should be informed to generally increase their physical activity (91.8%, 101/110), be prescribed exercises to target strengthening their calf-muscles (89.8%, 97/108), have a target for daily walking (80.8%, 88/109), and exercises targeting their ankle joint mobility (90.8%, 99/109) (see Figure 3).

The majority of clinicians (82.7% 86/104) were aware of clinical practice guidelines for managing VLUs such as Australian and New Zealand Clinical Practice Guideline for the Prevention and Management of Venous Leg Ulcer and guidelines produced by Scottish Intercollegiate Guidelines Network (SIGN). The majority of clinicians (78.4%; 80/102) reported that they were not specifically aware of evidence about prescribed exercises for adults with a VLU (eg, systematic reviews).

There were no differences to recommending PA based on age or number of years working in wound care (Mann Whitney U test p = 959 p = .548 respectively) or for recommending calf exercises (age p = .796, number of years p = .5). There was also no difference for recommending ankle mobility exercises based on clinician's age or number of years working in wound care (Mann Whitney p = .832, p = .667 respectively). However, there was a significant difference for recommending walking based on clinician's age (Mann Whitney U test p = .028) but not for number of years working in wound care (p = .714).

At the bivariate level of analysis, significant associations were found between clinicians’ self-efficacy, familiarity with exercises, and their likelihood to recommend PA interventions for adults with VLU. Self-efficacy was a strongly associated with recommending all exercise types. Clinicians with higher self-efficacy in prescribing calf muscle exercises were significantly more likely to recommend general PA increases, calf-strengthening exercises, walking, and ankle mobility exercises (p < .001). Self-efficacy in prescribing ankle exercises and walking was also highly associated with these exercise recommendations (p < .001). Familiarity with exercises followed a similar pattern, with familiarity of calf and ankle exercises significantly correlating with clinicians’ recommendation of general PA, calf-strengthening, walking, and ankle mobility exercises (p < .001). Awareness of evidence showed moderate associations with general PA (p = .007), calf-strengthening exercises (p = .015), and walking (p = .045), but no significant association with ankle mobility exercises (p = .341). Attitudes toward specific exercises had a mixed influence, with significant associations between agreement with calf muscle exercises and recommending them (p = .022), but attitudes toward walking and general PA showed weaker correlations. Outcome expectations were moderately associated with all types of PA recommendations, but particularly significant for calf-strengthening exercises (p = .017) and walking (p = .022). All bivariate results are displayed in Table 2.

**Table 2.** Relationships between attitudes, knowledge, and practices to understand the associations with barriers / enablers for promoting PA.

	Recommendation for exercise			
	General increase in physical activity	Calf-strengthening exercises	Walking to a target level of frequency and duration	Ankle mobility
Aware of evidence	.**007**	.015	.**045**	.341
**Confidence**				
Self-efficacy calf muscle exercises	. ***012***	***<***. ***001***	***<***. ***001***	***<***. ***001***
Self-efficacy ankle exercises	.090	***<***. ***001***	. ***001***	***<***. ***001***
Self-efficacy walking	. ***023***	. ***001***	***<***. ***001***	. ***007***
Self-efficacy exercise evidence	. ***003***	***<***. ***001***	***<***. ***001***	. ***003***
**Attitude**				
Agreement PA	.294	.075	.443	.296
Agreement calf muscle exercises	.342	.**022**	.282	1.0
Agreement walking	.**053**	.084	.093	.634
Agreement ankle exercises	. ***041***	. ***005***	.061	. ***019***
**Knowledge**				
Familiarity of calf muscle exercises	. ***008***	***<***. ***001***	***<***. ***001***	***<***. ***001***
Familiarity of walking	***<***. ***001***	. ***003***	***<***. ***001***	. ***032***
Familiarity of ankle exercises	. ***002***	***<***. ***001***	. ***011***	***<***. ***001***
Outcome Expectations	. ***037***	. ***017***	. ***022***	.202

The model −2 Log likelihood: 68.445, Cox and Snell R^2^= .523. Hosmer and Lemeshow test confirmed that the model was a good fit for the data, p = .587. Self-efficacy was a significant predictor of recommending ankle exercises (B = 2.032, p < .001, Exp(B) = 7.630 {95% CI: 3.568–16.317}), showing that clinicians with high confidence are 7.6 times more likely to recommend ankle exercises. Familiarity toward prescribing ankle mobility exercises for adults with VLUs in compression, though not statistically significant (B = .641, p = .087, Exp(B) = 1.898 {95% CI: .910–3.958}), may still contribute to clinical decision-making and warrants further exploration.

The free text “How do you decide which patients may need additional exercises and/or physical activity prescription?” was analysed thematically and three themes were identified:

1. Personalisation of Exercise Recommendations:

Healthcare professionals tailor exercise advice based on the functional abilities of each patient. For those with higher mobility, exercises like ankle stretches and walking are recommended, while immobile patients receive simpler tasks, such as ankle movements during TV commercials. This individualised approach ensures that exercises are achievable for all patients, emphasising safe and practical interventions, as described by one respondent:

They [patients] all get some form of encouragement, it depends on their current functional ability as to what is recommended. BUT I always ask for legs up for 20 min after the exercise as well. For patients that are not mobile at all I will often as them to do a series of ankle movements each time a commercial comes on the TV. Every patient is different.”

2. Holistic and Patient-Centred Care:

A holistic approach is common, considering both physical and psychological aspects of care. Clinicians assess patients’ mobility levels, independence, and overall well-being, often referring them to physiotherapists or providing educational materials. This comprehensive view addresses the healing of VLUs while enhancing quality of life, for example one respondent described.

“Holistic assessment to include current exercise levels. If moderate/low/none, advice booklets given out with ankle and calf exercises in. Fit and very active individuals are given verbal information as our booklets are more suited for less active individuals. Offered “well-being sessions” with sports therapist as part of our service, therapist is specially trained in lower limb exercised to target VLUs.”

3. Barriers to Exercise in older and Less Mobile Patients:

Age, comorbidities, and limited mobility are major barriers to engaging in exercise. Many older patients are not referred for physiotherapy, as clinicians are cautious about the risks. This highlights the need for alternative approaches and resources to encourage safe physical activity in frail populations as described by this respondent:

“Most of my patients are very elderly. No set criteria for referring to physio. Rarely refer them for exercise”

## Discussion

This study provides critical insights into clinicians’ knowledge, attitudes, and practices regarding PA for adults with VLUs. The findings reveal a significant gap between clinicians’ awareness of PA guidelines and their actual implementation in daily practice. Although 83% of respondents reported awareness of clinical practice guidelines for managing VLUs, only a small proportion consistently recommend specific PA interventions such as calf-strengthening exercises and ankle joint mobility exercises to their patients.

The study also identifies key barriers to guideline adherence, including a lack of training, access to exercise specialists, and resources for both clinicians and patients. These barriers contribute to the underutilisation of PA as a therapeutic strategy, despite its well-documented benefits for wound healing and overall quality of life in patients with a VLU. These barriers are consistent with prior research, which highlights the challenges of embedding PA into wound care management.^15^ Despite evidence showing that PA can improve wound healing and overall patient quality of life,^11^ a lack of confidence and structured support may prevent clinicians from advocating for these interventions regularly.

The results also indicate that while clinicians generally perceive themselves as knowledgeable about specific exercises for VLUs, their self-efficacy varies considerably. For instance, 66.1% reported at least adequate familiarity with ankle mobility exercises, yet only 59% felt moderately confident in discussing the evidence supporting exercise with their patients. This discrepancy suggests that while awareness exists, there may be a need for more practical training or resources to bridge the gap between knowledge and practice. Many studies emphasise the critical role of healthcare providers in facilitating patient adherence to exercise regimens for chronic conditions.^24,25^

Interestingly, clinicians with higher self-efficacy were 6 times more likely to recommend ankle mobility exercises, underscoring the importance of confidence in influencing PA prescription. This points to a potential intervention area: enhancing clinician training could significantly improve the frequency of exercise recommendations. Promoting PA is essential for enhancing health and well-being in adults generally,^26^ including those with VLUs. During follow-up treatment visits, clinicians have a unique opportunity to encourage lifestyle changes that promote physical activity among patients.^27^ The predominance of nurses in our sample likely reflects their central role in venous leg ulcer management in many healthcare systems. However, this focus may overlook important perspectives from other healthcare professionals, such as physicians, physiotherapists, and allied health staff, who may have differing approaches or priorities in promoting PA.

Clinicians emphasised tailoring exercise prescriptions to patients’ mobility levels, suggesting that while most patients received some form of PA recommendation, the specific interventions varied based on their functional capacity. This personalised approach is critical in managing a heterogeneous patient population, where one-size-fits-all recommendations may not be effective. However, clinicians face challenges when working with older, less mobile patients. Barriers such as age-related comorbidities and limited mobility reduce the likelihood of recommending more intensive PA regimens, such as walking. This highlights the need for alternative strategies, such as modified, low-intensity exercises, that can be safely incorporated into the care of frail populations.

## Implications for Clinical Practice

The findings suggest that addressing the systemic barriers clinicians face, such as lack of access to exercise specialists and relevant patient resources, is crucial for improving adherence to PA guidelines. Providing targeted training that enhances self-efficacy in recommending PA, as well as developing comprehensive resources for both clinicians and patients, could bridge the gap between knowledge and practice.

Furthermore, large-scale implementation strategies, could incorporate structured approaches such as the VaLUE (Venous Leg Ulcers and Exercise) program^28^ which is specifically designed for adults with VLUs to strengthen the calf muscle pump, support venous return and enhance ankle mobility, may be translated into practical frameworks that clinicians can easily adopt.^29^ Future interventions should focus on tailoring exercise prescriptions to suit the diverse needs of patients with a VLU, particularly those with mobility limitations, while ensuring that clinicians have the necessary support to promote these interventions confidently.

## Limitations

This study's findings may have limited generalisability, given that 99% of respondents were nurses, which may not represent the perspectives of other healthcare providers involved in VLU management. The predominance of nurses in our sample and the limited geographical distribution (primarily the UK and Australia) significantly constrain the generalisability of our findings to other healthcare professionals and diverse healthcare systems, highlighting the need for future research to include a broader and more representative sample. Our study is limited by the absence of formal validation for the survey instrument, a high proportion of missing data and the use of convenience sampling. While missing data were addressed by analysing only valid responses, this approach may have introduced bias. Moreover, the reliance on convenience sampling could overrepresent participants with a specific interest in wound care, underscoring the need for future studies to incorporate rigorous survey validation and stratified sampling techniques. Potential confounding variables, such as the clinician's years of experience, professional role, access to training, and workplace setting (eg, rural vs urban), were considered during data interpretation. While these factors may influence clinicians’ knowledge, attitudes, and practices, the study did not include detailed subgroup analyses to isolate their effects. Future research could further explore these variables to identify their specific contributions and strengthen the evidence base for physical activity promotion in venous leg ulcer management.

## Conclusion

This research is a critical first step in understanding barriers and enablers for clinicians to promote exercise for adults with VLUs. This study highlights a need to address clinician training and resources to promote PA among adults with VLUs. Although the benefits of PA are well-documented, systemic barriers hinder consistent guideline adherence. By enhancing clinician confidence and providing tailored interventions, we can potentially improve VLU healing outcomes and overall patient well-being.

## Supplemental Material

sj-docx-1-ijl-10.1177_15347346251321550 - Supplemental material for The Role of Clinicians’ Self-Efficacy in Exercise Promotion for Adults with Venous Leg Ulcers: A Cross-Sectional Study Supplemental material, sj-docx-1-ijl-10.1177_15347346251321550 for The Role of Clinicians’ Self-Efficacy in Exercise Promotion for Adults with Venous Leg Ulcers: A Cross-Sectional Study by Jane O’Brien, Damhnat McCann, Christina N Parker, Kathleeen Finlayson and Andrew Jull in The International Journal of Lower Extremity Wounds

sj-docx-2-ijl-10.1177_15347346251321550 - Supplemental material for The Role of Clinicians’ Self-Efficacy in Exercise Promotion for Adults with Venous Leg Ulcers: A Cross-Sectional Study Supplemental material, sj-docx-2-ijl-10.1177_15347346251321550 for The Role of Clinicians’ Self-Efficacy in Exercise Promotion for Adults with Venous Leg Ulcers: A Cross-Sectional Study by Jane O’Brien, Damhnat McCann, Christina N Parker, Kathleeen Finlayson and Andrew Jull in The International Journal of Lower Extremity Wounds

## References

[1] Persoon A Heinen MM Van Der Vleuten CJ De Rooij MJ Van De Kerkhof PC Van Achterberg T . Leg ulcers: A review of their impact on daily life. J Clin Nurs. 2004;13(3):341-354. 15009337 10.1046/j.1365-2702.2003.00859.x

[2] Posnett J Gottrup F Lundgren H Saal G . The resource impact of wounds on health-care providers in Europe. J Wound Care. 2009;18(4):154-154. 19349935 10.12968/jowc.2009.18.4.41607

[3] Agale SV . Chronic leg ulcers: epidemiology, aetiopathogenesis, and management. Ulcers. 2013;2013:1.

[4] Finlayson K Wu M-L Edwards HE . Identifying risk factors and protective factors for venous leg ulcer recurrence using a theoretical approach: A longitudinal study. Int J Nurs Stud. 2015;52(6):1042-1051. 25801312 10.1016/j.ijnurstu.2015.02.016

[5] Abbade LPF Lastória S de Almeida Rollo H Ometto Stolf H . A sociodemographic, clinical study of patients with venous ulcer. Int J Dermatol. 2005;44(12):989-992. 16409260 10.1111/j.1365-4632.2004.02276.x

[6] Saha SP Whayne TF Mukherjee D . Current management of peripheral vascular disease: Where is the evidence? Cardiovascular & Hematological Agents in Medicinal Chemistry (Formerly Current Medicinal Chemistry-Cardiovascular & Hematological Agents). 2011;9(2):128-136. 10.2174/187152511796196489 21434864

[7] Weller C Richards C Turnour L Team V . Venous leg ulcer management in Australian primary care: Patient and clinician perspectives. Int J Nurs Stud. 2021;113:103774. 33080480 10.1016/j.ijnurstu.2020.103774

[8] Jull A Muchoney S Parag V Wadham A Bullen C Waters J . Impact of venous leg ulceration on health-related quality of life: A synthesis of data from randomized controlled trials compared to population norms. Wound Repair Regen. 2018;26(2):206-212. 30015365 10.1111/wrr.12636

[9] Wounds Australia. Standards for wound prevention and management. Wounds Australia; 2016:0980739691.

[10] Kelechi TJ Brunette G Bonham PA , et al. 2019 Guideline for management of wounds in patients with Lower-Extremity Venous Disease (LEVD): An executive summary. J Wound Ostomy Continence Nurs. 2020;47(2):97-110. doi:https://doi.org/10.1097/WON.0000000000000622 32150136 10.1097/WON.0000000000000622

[11] Jull A Slark J Parsons J . Prescribed exercise with compression vs compression alone in treating patients with venous leg ulcers: A systematic review and meta-analysis. JAMA Dermatol. 2018;154(11):1304-1311. 30285080 10.1001/jamadermatol.2018.3281 PMC6248128

[12] Kelechi TJ Brunette G Bonham PA Crestodina L . 2019 Guideline for management of wounds in patients with Lower Extremity Venous Disease (LEVD): An executive summary. Journal of Wound, Ostomy & Continence Nursing. 2020;47(2):97-110. doi:10.1097/WON.0000000000000622 32150136

[13] Neumann H Cornu-Thenard Junger M , et al. Evidence-based (S3) guidelines for diagnostics and treatment of venous leg ulcers. J Eur Acad Dermatol Venereol. 2016;30(11):1843-1875. doi:10.1111/jdv.13848 27558268

[14] Wounds UK . Best Practice Statement: Holistic management of venous leg ulceration. Wounds UK; 2016.

[15] Qiu Y Team V Osadnik CR Weller CD . Barriers and enablers to physical activity in people with venous leg ulcers: A systematic review of qualitative studies. Int J Nurs Stud. 2022;135:104329. 35986960 10.1016/j.ijnurstu.2022.104329

[16] Demark-Wahnefried W Aziz NM Rowland JH Pinto BM . Riding the crest of the teachable moment: Promoting long-term health after the diagnosis of cancer. Journal of Clinical Oncology: Official Journal of the American Society of Clinical Oncology. 2005;23(24):5814. 16043830 10.1200/JCO.2005.01.230 PMC1550285

[17] Daley AJ Bowden SJ Rea DW Billingham L Carmicheal AR . What advice are oncologists and surgeons in the United Kingdom giving to breast cancer patients about physical activity? International Journal of Behavioral Nutrition and Physical Activity. 2008;5(1):46. 18803812 10.1186/1479-5868-5-46 PMC2553795

[18] Qiu Y Osadnik CR Team V . Planning exercise interventions as an adjunct treatment in managing venous leg ulcers: A qualitative study of nurses’ perspectives. J Tissue Viability. 2023;32(2):279-285. 37032305 10.1016/j.jtv.2023.03.004

[19] Monkey. S. Create surveys. Get answers. 2009.

[20] Von Elm E . STROBE initiative. The strengthening the reporting of observational studies in epidemiology (STROBE) statement: Guidelines for reporting observational studies. Ann Intern Med. 2007;147:573-577. 17938396 10.7326/0003-4819-147-8-200710160-00010

[21] Cabana MD Rand CS Becher OJ Rubin HR . Reasons for pediatrician nonadherence to asthma guidelines. Arch Pediatr Adolesc Med. 2001;155(9):1057-1062. 11529809 10.1001/archpedi.155.9.1057

[22] Pathman DE Konrad TR Freed GL Freeman VA Koch GG . The awareness-to-adherence model of the steps to clinical guideline compliance: The case of pediatric vaccine recommendations. Med Care. 1996;873-889. 8792778 10.1097/00005650-199609000-00002

[23] Clarke V Braun V . Thematic analysis. J Posit Psychol. 2017;12(3):297-298.

[24] Daley A . Exercise and depression: A review of reviews. J Clin Psychol Med Settings. 2008;15:140-147. 19104978 10.1007/s10880-008-9105-z

[25] Demark-Wahnefried W Aziz NM Rowland JH Pinto BM . Riding the crest of the teachable moment: Promoting long-term health after the diagnosis of cancer. J Clin Oncol. 2005;23(24):5814-5830. 16043830 10.1200/JCO.2005.01.230 PMC1550285

[26] Bull FC Al-Ansari SS Biddle S , et al. World Health Organization 2020 guidelines on physical activity and sedentary behaviour. Br J Sports Med. 2020;54(24):1451-1462. 33239350 10.1136/bjsports-2020-102955 PMC7719906

[27] Parker NH Arlinghaus KR Johnston CA . Integrating physical activity into clinical cancer care. Am J Lifestyle Med. 2018;12(3):220-223. doi:10.1177/1559827618759478 30283253 PMC6124961

[28] O’Brien JA Finlayson KJ Kerr G Edwards HE . Testing the effectiveness of a self-efficacy based exercise intervention for adults with venous leg ulcers: Protocol of a randomised controlled trial. BMC Dermatol. 2014;14:1-9. 25277416 10.1186/1471-5945-14-16 PMC4188410

[29] O'Brien J Finlayson K Kerr G Edwards H . Evaluating the effectiveness of a self-management exercise intervention on wound healing, functional ability and health-related quality of life outcomes in adults with venous leg ulcers: A randomised controlled trial. Int Wound J. 2017;14(1):130-137. 26817648 10.1111/iwj.12571 PMC7949716

