# Supplementary material for: The Role of Clinicians’ Self-Efficacy in Exercise Promotion for Adults with Venous Leg Ulcers: A Cross-Sectional Study
Source: Int J Low Extrem Wounds. 2025 Feb 25;25(3):979–87. doi: 10.1177/15347346251321550 (PMC13438010; doi:10.1177/15347346251321550)
Supplement: Supplementary file 1 [file sj-docx-1-ijl-10.1177_15347346251321550.docx]

**Supplementary File**

**Table A1 List of all questions included in the survey with corresponding answers.**

| **Statement** | **Response** | **Count (%)** |
| --- | --- | --- |
| ***Please indicate your familiarity (KNOWLEDGE) with the following aspects of exercises for VLU management:*** | | |
| Types of simple exercises that increase ankle joint mobility | Extremely familiar | 14 (12.5%) |
|  | Very familiar | 29 (25.9%) |
|  | Adequately familiar | 31 (27.7%) |
|  | Somewhat familiar | 26 (23.2%) |
|  | Not at all familiar | 18 (10.7%) |
| Types of simple exercises that increase calf-muscle strength | Extremely familiar | 12 (10.6%) |
|  | Very familiar | 22 (19.5%) |
|  | Adequately familiar | 33 (29.2%) |
|  | Somewhat familiar | 34 (30.1%) |
|  | Not at all familiar | 12 (10.6%) |
| The duration and frequency of walking for which VLU patients should aim | Extremely familiar | 5 (4.5%) |
|  | Very familiar | 21 (18.8%) |
|  | Adequately familiar | 24 (21.4%) |
|  | Somewhat familiar | 27 (24.1%) |
|  | Not at all familiar | 35 (31.3%) |
| ***Please rate your confidence (SELF-EFFICACY) in your ability to:*** | | |
| Demonstrate and prescribe ankle joint mobility exercises for a VLU patient | Extremely confident | 21 (17.9%) |
|  | Very confident | 36 (30.8%) |
|  | Moderately confident | 23 (19.7%) |
|  | Somewhat confident | 19 (16.2%) |
|  | Not at all confident | 18 (15.4%) |
| Demonstrate and prescribe calf-muscle strengthening exercises for a VLU patient | Extremely confident | 18 (15.4%) |
|  | Very confident | 27 (23.1%) |
|  | Moderately confident | 37 (31.6%) |
|  | Somewhat confident | 15 (12.8%) |
|  | Not at all confident | 20 (17.1%) |
| Build towards a targeted increase in walking in an inactive VLU patient | Extremely confident | 8 (6.9%) |
|  | Very confident | 17 (14.7%) |
|  | Moderately confident | 30 (25.9%) |
|  | Somewhat confident | 39 (33.6%) |
|  | Not at all confident | 22 (19%) |
| Discuss the evidence for exercises with VLU patients | Extremely confident | 13 (11.1%) |
|  | Very confident | 25 (21.4%) |
|  | Moderately confident | 31 (26.5%) |
|  | Somewhat confident | 27 (23.1%) |
|  | Not at all confident | 21 (17.9%) |
| ***How significantly does each factor influence how frequently you would recommend adding exercises for treating VLU patients in compression?*** | | |
| Lack of training | Extremely significant | 44 (40%) |
|  | Moderately significant | 33 (30%) |
|  | Slightly significant | 21 (19.1%) |
|  | Not at all significant | 12 (10.9%) |
| Lack of information for your use | Extremely significant | 41 (37.3%) |
|  | Moderately significant | 42 (38.2%) |
|  | Slightly significant | 16 (14.5%) |
|  | Not at all significant | 11 (10%) |
| Lack of information for patient use | Extremely significant | 50 (45.9%) |
|  | Moderately significant | 39 (35.8%) |
|  | Slightly significant | 13 (11.9%) |
|  | Not at all significant | 7 (6.4%) |
| Lack of inhouse exercise specialists | Extremely significant | 53 (48.2%) |
|  | Moderately significant | 30 (27.3%) |
|  | Slightly significant | 17 (15.5%) |
|  | Not at all significant | 10 (9.1%) |
| Lack of other services to refer patients | Extremely significant | 46 (41.8%) |
|  | Moderately significant | 36 (32.7%) |
|  | Slightly significant | 19 (17.3%) |
|  | Not at all significant | 9 (8.2%) |
| ***Please indicate if you agree or disagree with the following:*** | | |
| VLU patients in compression should be informed to generally increase their physical activity | Strongly agree | 47 (42.7%) |
|  | Agree | 54 (49.1%) |
|  | Neither agree nor disagree | 7 (6.4%) |
|  | Strongly disagree | 2 (1.8%) |
| VLU patients in compression should be prescribed exercises to target strengthening their calf-muscles | Strongly agree | 42 (38.9%) |
|  | Agree | 55 (50.9%) |
|  | Neither agree nor disagree | 9 (8.3%) |
|  | Strongly disagree | 2 (1.9%) |
| VLU patients in compression should be prescribed a target for daily walking | Strongly agree | 39 (35.8%) |
|  | Agree | 49 (45%) |
|  | Neither agree nor disagree | 20 (18.3%) |
|  | Strongly disagree | 1 (0.9%) |
| VLU patients in compression should be prescribed exercises targeting their ankle joint mobility | Strongly agree | 51 (46.8%) |
|  | Agree | 48 (44%) |
|  | Neither agree nor disagree | 8 (7.3%) |
|  | Strongly disagree | 2 (1.8%) |
| **Based on your experience with VLU patients in compression, what is the effect on healing of patients doing prescribed exercises?** | Large effect | 27 (26.2) |
|  | Moderate effect | 58 (56.3) |
|  | Little effect | 17 (16.5) |
|  | No effect | 1 (1) |
| **AWARENESS: Please indicate yes or no for the following:** | | |
| Are you aware of recent evidence about prescribed exercises for VLU? (e.g. systematic reviews) | Yes | 22 (21.6) |
|  | No | 80 (78.4) |
| Prior to this survey were you aware of any clinical practice guidelines for managing VLU? (e.g. AWMA, SIGN, NICE) | Yes | 86 (82.7) |
|  | No | 18 (17.3) |
